# Effects of the healing beats application on anxiety, stress, and well-being in hemodialysis patients in Korea: a randomized controlled trial

**DOI:** 10.7586/jkbns.24.042

**Published:** 2025-02-24

**Authors:** Se-Na Yu, Myung-Haeng Hur

**Affiliations:** 1 Nowon Eulji University Hospital, Seoul, Korea; 2 College of Nursing, Eulji University, Uijeongbu, Korea

**Keywords:** Anxiety, Stress, Psychological well-being, Renal dialysis, Music therapy

## Abstract

**Purpose:**

This study examined the effects of Healing Beats on anxiety, stress, and well-being in hemodialysis patients, exploring its potential as an intervention to improve these outcomes.

**Methods:**

A randomized controlled trial included 65 hemodialysis patients with chronic kidney disease, divided into the Healing Beats group (HBG, 21 participants), preferred music group (PMG, 22 participants), and control group (CG, 22 participants). The HBG was exposed to music synchronized with their heartbeats during each dialysis session, while the PMG listened to music of their own selection. The CG received no music intervention. State anxiety, subjective stress, stress indices, and well-being were assessed before and after the experimental intervention.

**Results:**

The application of Healing Beats had a positive effect on the stress index of hemodialysis patients, with statistically significant differences between the three groups (F = 4.60, *p* < .001). However, no significant differences were observed in terms of anxiety and well-being across the groups after the intervention.

**Conclusion:**

Although hemodialysis patients have notably high stress levels, the application of Healing Beats and preferred music demonstrated limitations in alleviating anxiety and enhancing well-being. Although Healing Beats proved effective in reducing the stress index, it did not yield significant improvements in subjective stress, anxiety, or well-being. Further research is required to confirm these findings and explore potential refinements in the intervention.

## INTRODUCTION

Hemodialysis is a procedure performed to prevent and treat uremia in patients whose renal function has been temporarily or permanently impaired [1]. As of 2023, the total number of patients receiving renal replacement therapy in South Korea is 137,705, with 110,443 undergoing hemodialysis, 5,253 receiving peritoneal dialysis, and 22,009 having undergone kidney transplants. Notably, hemodialysis accounts for 83.7% of the total, representing the highest proportion of renal replacement therapy [2]. The number of hemodialysis patients, which was approximately 20,000 in 2002, has increased nearly sevenfold, with 18,052 new cases reported in 2023 alone [2].

In hemodialysis, patients undergo three sessions per week, each lasting 4 hours [3]. For patients, dialysis represents the only viable option for survival, as the underlying disease is incurable. The long-term dependence on dialysis and the fear of death often lead to profound emotional distress, resulting in depression and despair [4]. Stressors such as illness can disrupt the sense of life’s meaning, leading to emotional turmoil, helplessness, internal emptiness, and feelings of worthlessness [5]. These negative emotional states, including depression, anxiety, and stress, adversely impact the treatment outcomes and overall quality of life of hemodialysis patients [6]. Chronic kidney disease, categorized as a non-cancerous terminal illness, has been included in the "Act on Hospice and Palliative Care and Decisions on Life-Sustaining Treatment for Patients at The End of Life" since April 2018 [7].

Life-long dependence on dialysis machines, coupled with the fear of complications, significantly influences the treatment outcomes and quality of life of dialysis patients [8]. Given the rapid increase in the number of hemodialysis patients in South Korea [2], there is an urgent need to explore effective nursing interventions to address the growing demands and needs for stress management in this patient population.

A meta-analysis of previous studies has suggested that stress intervention methods, including aromatherapy, music therapy, humor, hand massage, and meditation, have a moderate effect in reducing psychological stress in hemodialysis patients. Regarding physiological stress, the application of stress management programs led to a statistically significant reduction in stress levels in the experimental group compared to the control group (CG). When analyzed by intervention type, aromatherapy and music therapy showed significant differences in standardized mean differences compared to the CG [8]. Other studies also reported that music therapy was effective in reducing psychological stress [9]. Regarding the duration of the interventions for dialysis patients, both subgroups of interventions lasting 60 minutes or less, and those exceeding 60 minutes, were found to be effective in a subgroup analysis. Additionally, interventions with fewer than 10 sessions, between 10 and 20 sessions, and more than 20 sessions all showed effectiveness [8].

Music has been shown to influence metabolism, vital signs, and even block sounds that induce anxiety. One of the advantages of music interventions is that they are cost-effective and can be independently administered by nurses [10,11]. Moreover, prior research has demonstrated that music interventions have overall positive effects on improving quality of life [12]. Music has been shown to improve the well-being and quality of life of chronic patients [13]. To optimize the effects of music therapy, it is crucial to consider the participant’s sex, past experiences, familiarity with music, music preferences, as well as mood and cultural resonance [14]. This suggests that music should not be chosen solely based on preference but should be tailored to reflect individual characteristics [15].

Recently, a music intervention known as Healing Beats has been introduced, which applies a beats-per-minute (bpm) frequency matching the individual’s resting heart rate, promoting autonomic balance and inducing relaxation. This method has been shown to reduce anxiety and stress in nursing students [16] and cancer patients undergoing chemotherapy [17]. Although there are limited studies investigating the application of music interventions in hemodialysis patients, most of the existing studies have utilized classical music or patient-preferred music [8,18]. Additionally, studies specifically examining the effects of music therapy on anxiety, stress, and well-being in this patient population are scarce. And in dialysis patients vulnerable to anxiety and stress, most studies only evaluate negative emotions [19], and few studies have evaluated whether they achieve healthy adaptation by positively accepting negative aspects.

Healing Beats is a patented sound intervention developed in South Korea, yet its effects on well-being have not been previously studied. However, well-being is considered a critical factor in achieving therapeutic goals for hemodialysis patients [20]. Based on clinical experience, enhancing the sense of well-being in this patient population is recognized as essential, which led to its selection as a key variable in this study. Furthermore, Healing Beats, as a sound-based intervention, can be regarded as a form of music therapy. Supporting evidence includes studies indicating that music or therapeutic sounds positively influence the well-being of individuals [21]. Additionally, a randomized controlled trial demonstrated that music therapy significantly enhances well-being in palliative care settings [22], with a partial eta squared value of 0.07. These findings provide a foundation for investigating the potential effects of Healing Beats in this context.

Therefore, this study aims to investigate the impact of Healing Beats, a music-based intervention utilizing bpm matching an individual’s heart rate, on anxiety, stress, and well-being in hemodialysis patients. By exploring this, the study seeks to assess how Healing Beats can modify these psychological outcomes in patients undergoing hemodialysis.

## METHODS

### 1. Study design

**Figure 1.**
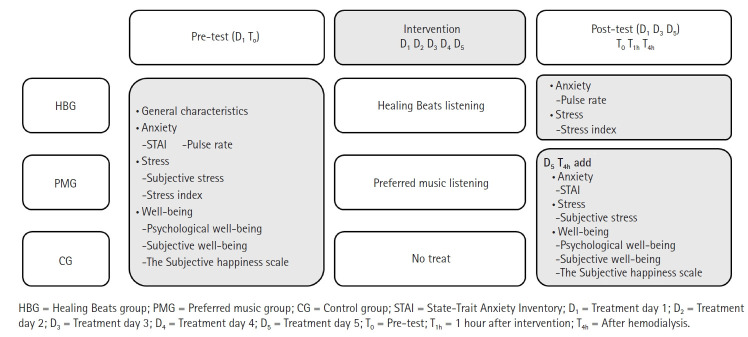
Overview of the study design and methodology.

This study utilized a equivalent CG pretest-posttest experimental design to investigate the effects of Healing Beats on anxiety, stress, and well-being in hemodialysis patients (Figure 1).

### 2. Participants

The study was conducted among adults who were diagnosed with chronic renal failure and undergoing hemodialysis treatment at Nowon Eulji Medical Center in Seoul city. Participants were recruited based on an advertisement posted on the dialysis unit's bulletin board.

The inclusion criteria were as follows: adults aged 20 years or older who had been diagnosed with chronic renal failure for at least 3 months and were undergoing hemodialysis three times a week on an outpatient basis. Participants had to have no hearing impairments that would interfere with music listening, be capable of understanding the purpose of the study and communicating effectively, and provide consent to participate in the research. The exclusion criteria were as follows: individuals who were hospitalized, those with unclear consciousness or disorientation, those currently taking medications that could affect anxiety or stress (such as anxiolytics or sedatives), individuals with a history of auditory disorders, or those currently suffering from auditory conditions.

The sample size was determined using the G*Power 3.1.9.7 [23] program by inputting the alpha level, power, and effect size. Based on a previous study [24] that investigated the effects of Healing Beats on stress, with an effect size of 0.28, the same effect size was applied in this study. For the repeated measures analysis of variance (ANOVA) of the three groups, with a significance level of 0.05, a power (1-β) of 0.95, three measurements, and a correlation of 0.3 [25] between repeated measures, the required sample size was calculated to be 20 participants in each of the Healing Beats, preferred music, and CGs. Considering a 10% dropout rate, 22 participants were randomly assigned to each group. However, one participant in the Healing Beats group (HBG) was hospitalized due to health deterioration during the experiment, leaving 21 participants in the HBG, 22 in the preferred music group (PMG), and 22 in the CG, resulting in a total of 65 participants whose data were used in the analysis.

**Figure 2.**
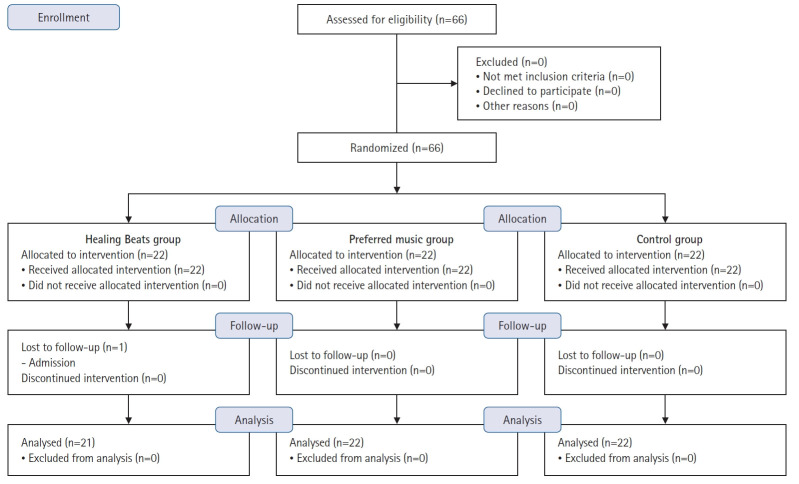
Study flow diagram.

After gathering participant lists through recruitment notices, participants were randomly assigned to one of three groups (Healing Beats, preferred music, or control) using a random number generator in Microsoft Excel. To prevent information exchange, the Healing Beats and PMGs were assigned to Monday-Wednesday-Friday sessions, while the CG was assigned to Tuesday-Thursday-Saturday sessions. Participants were not informed about which group they were assigned to (Figure 2).

### 3. Measurements

**1) *Anxiety*:** anxiety is a subjective emotional state that changes according to the situation, characterized by tension or worry that is consciously perceived. It is a temporary emotional state that occurs when an individual perceives an environment as threatening, regardless of objective threat [26]. It was measured by objective and subjective indicators [24].

**(1) Anxiety measurement:** anxiety was measured using the State-Trait Anxiety Inventory developed by Spielberger [26], with the state anxiety items adapted for Korean characteristics by Kim and Shin [27]. The tool consists of 10 positive and 10 negative items, with a 4-point Likert scale. For the positive items, reverse scoring was applied. The score ranges from 20 to 80, with higher scores indicating higher levels of anxiety. In Kim and Shin’s [27] study, Cronbach’s α was .87, while in the current study, Cronbach’s α was .91.

**(2) Vital signs:** in this study, pulse rate was measured using the automatic blood pressure monitor (FMC 5008S, Fresenius Medical Care, German; Phoenix, Baxter, Illinois, USA; Artis, Baxter, Illinois, USA), with the measurement taken from the upper arm opposite the arteriovenous fistula after the participant had rested.

**2) *Stress*:** stress is a specific syndrome composed of all changes that occur non-specifically within the body [28]. It was measured by objective and subjective indicators [8,25].

**(1) Stress index:** using the autonomic nervous system measurement device Canopy9 professional 4.0 (IEMBIO, Gangwon-do, Korea), stress index was measured for 5 minutes. The stress level was quantified based on the balance of autonomic nervous system, which is derived from heart rate variability (HRV) using standard lead methods. The values range from 1 to 10, with higher numbers indicating greater exposure to stressful situations.

**(2) Subjective stress:** subjective stress was assessed using the Numeric Rating Scale, which assigns a score from 0 to 10, with higher scores indicating greater stress.

**3) *Well-Being*:** this term emphasizes an individual's perception or overall feeling, referring to self-assessment of physical health and general feelings about physical fitness [29]. Life satisfaction, emotions, and life expectations were measured [30,31].

**(1) Psychological well-being:** the tool used was Ryff's [32] psychological well-being, adapted by An [33]. The scale consists of 18 items across six subdomains: autonomy, environmental mastery, personal growth, purpose in life, positive relations with others, self-acceptance. The scale uses a 5-point Likert format, with scores ranging from 18 to 90. A higher score indicates higher psychological well-being. In previous studies, Cronbach’s α was .84 [33], and in this study, it was .68.

**(2) Subjective well-being:** the subjective emotion frequency scale, originally developed by Zoh and Cha [34], and later revised by Kim [30], was utilized. This scale assesses subjective emotional states through four positive and four negative emotions, each rated on a 7-point Likert scale. The total score ranges from 0 to 56, with higher scores indicating greater levels of positive emotion. In previous studies, the Cronbach's α for negative emotions was .73, and for positive emotions, it was .63 [30]. In this study, the Cronbach's α for negative emotions was .77, and for positive emotions, it was .76.

**(3) The subjective happiness scale (SHS):** developed by Lyubomirsky and Lepper [35] and adapted by Kim [30] was also used. This scale consists of four items related to happiness, rated on a 7-point Likert scale. The total score ranges from 0 to 28, with higher scores indicating greater levels of subjective happiness. The Cronbach's α for the SHS was reported as .76 in previous studies [30], while the current study yielded a reliability coefficient of .67.

### 4. Experiment

**1) Experimental environment:** all procedures followed coronavirus disease 2019 safety guidelines. The temperature of the experimental room was maintained at 22°C-24°C, and humidity was kept between 40%-60%. Participants in the experimental groups wore wireless earphones (T1C, QCY, China) and controlled the volume on their smartphones to a comfortable level for listening to music.

**2) Experimental intervention:** the experimental intervention in this study involved either listening to Healing Beats or preferred music.

(1) Healing Beats (Patent Registration No. 1021629180000; inventors: Baek Ik-Ryul, Hur Myung-Haeng) is a stress-relief system that utilizes heart rate-synchronized audio tracks based on research suggesting that beat induction and music beats can influence human heartbeats. It comprises a collection of 41 audio tracks specifically designed with rhythms aligned to normal heart rate ranges (60–100 bpm) at a steady tempo of one beat per second (quarter note in 4/4 time signature). The tracks simulate the sinus rhythm P-QRS-T wave form by analyzing variations in the power spectrum of electrocardiograms and extracting sound waves with frequency and amplitude similar to the participant’s resting heart rate. These repetitive rhythmic patterns include beat induction produced by piano and guitar [20]. In this study, the intervention involved applying a Healing Beats track synchronized to the participant's resting heart rate immediately before the start of hemodialysis. The audio tracks were played via a smartphone application and delivered through earphones for 1 hour at a comfortable volume suitable for the participant.

(2) Preferred music, on the other hand, refers to the therapeutic use of music to restore, maintain, or enhance physical and mental health [36]. Participants selected four tracks from a curated playlist of popular songs tailored to their age and preferences, prepared from major domestic music platforms (e.g., Melon, Genie, Naver Music). If there were no preferred music in the playlist, additional music were requested and selected.

(3) The selected tracks were played on a loop via a smartphone and earphones at a comfortable volume during the first hour of dialysis. For the experimental groups, participants in the HBG listened to tracks aligned to their heart rate, while those in the PMG listened to their selected tracks.

(4) The CG received no auditory intervention and remained in a relaxed state.

The intervention was applied 5 times over 10 days during consecutive dialysis visits. Based on prior research in music therapy [12,37] and with hemodialysis patients [8], the music intervention was standardized to 1 hour per session.

**3) Experimental procedures:** the experimental procedure was structured into three main phases: pre-intervention assessment, intervention, and post-intervention assessment. The detailed experimental procedures were as follows:

**(1) Pre-intervention assessment:** upon arrival at the dialysis unit, participants completed a pre-intervention questionnaire to measure anxiety, stress, and well-being. Stress index was assessed, followed by blood pressure and heart rate measurements. For the HBG, the smartphone application was installed, and participants were provided instructions on its operation. For the PMG, participants selected music from a curated playlist. The selected tracks were downloaded via Genie Music (www.genie.co.kr) and transferred to participants’ smartphones via Bluetooth, with an explanation ensuring the files were not distributed for purposes outside the study.

**(2) Experimental intervention (day 1–day 5);**

• HBG: participants listened to Healing Beats synchronized to their heart rate for 1 hour from the start of dialysis (T_1h_).

• PMG: participants listened to four selected songs on repeat for 1 hour via their smartphone (T_1h_).

• CG: participants remained in a relaxed and comfortable position without any auditory intervention during dialysis.

The intervention was conducted during five consecutive dialysis sessions over a 10-day period. Each session lasted for 1 hour, as suggested by prior studies on music therapy [12,37] and interventions for hemodialysis patients [8].

**(3) Post-intervention assessment:** to confirm the effect, measurements were made before and after the start date of the study [19,38], the third dialysis session of the intervention [39], and the end date of the study [38]. Additionally, music was provided for 1 hour at the start of dialysis, which was a stressor, and then measurements were taken [19,38].

**• First post-intervention assessment (day 1):** after the intervention, stress index, blood pressure, and heart rate were measured at 1 hour (T_1h_) and 4 hours (T_4h_) post-intervention using the Canopy9 Professional 4.0 system (IEMBIO, Gangwon-do, Korea). For the CG, the same measurements were taken at corresponding time intervals based on dialysis start time.

**• Second post-intervention assessment (day 3):** on the third dialysis session, pre-dialysis (T_0_), and 1 hour (T_1h_) and 4 hours (T_4h_) post-intervention, blood pressure, heart rate, and stress index were assessed. The CG underwent the same assessment schedule relative to dialysis start time.

**• Third post-intervention assessment (day 5):** during the fifth dialysis session, pre-dialysis (T_0_) and 1 hour (T_1h_) and 4 hours (T_4h_) post-intervention, blood pressure, heart rate, and stress index were measured. Additionally, participants completed a post-intervention questionnaire assessing anxiety, stress, and well-being. The CG followed the same measurement schedule relative to dialysis start time.

**4) Research assistant preparation:** one research assistant, a nurse with over 5 years of clinical experience, received 1 hour of prior training on smartphone application operation and Bluetooth earphone usage. The assistant provided support to participants for completing surveys and managing music playback if needed. To maintain blinding, the research assistant was not briefed on the study’s protocol or objectives.

### 5. Data Collection

Data were collected from July 19 to July 30, 2021, after approval from the Institutional Review Board (IRB) at Nowon Eulji Medical Center. The process involved recruiting participants through notices, obtaining consent, random assignment of participants, and data coding. Participants were provided with small tokens of appreciation after the study was completed.

### 6. Data Analysis

Data were analyzed using SPSS version 26.0 (IBM Corp., Armonk, NY, USA). Descriptive statistics (frequency, percentage) were used to analyze demographic characteristics. Homogeneity tests among the three groups were conducted using the chi-square test and ANOVA. Differences in anxiety, subjective stress, and well-being before and after the intervention were analyzed using ANOVA. ANOVA, repeated measures ANOVA, and repeated measures analysis of covariance (ANCOVA) were used to assess changes in heart rate and stress index over time. Additionally, the stress index was analyzed using a linear mixed effects model.

### 7. Ethical Considerations

All ethical guidelines, including voluntary participation, confidentiality, and informed consent, were adhered to throughout the study. Approval for this study was obtained from the IRB of the Nowon Eulji Medical Center following a comprehensive review of the study protocol (IRB No. EMC 2021-05-023-002). Written informed consent was secured from all participants voluntarily agreed, who were explicitly informed of their right to withdraw from the study at any time without penalty. All data collected during the study were anonymized and securely stored to ensure confidentiality. Upon completion of the experiment, participants received a token of appreciation in the form of earphones. Study-related data will be retained securely for three years after the conclusion of the research and will then be disposed of using a document shredder to maintain data integrity. Finally, upon request, participants in the CG were granted access to the Healing Beats program after the study’s completion.

## RESULTS

### 1. Homogeneity test of general characteristics and dependent variables

This study included a total of 65 participants: 21 in the HBG, 22 in the PMG, and 22 in the CG. Regarding general characteristics, the sex distribution was as follows: 9 men (42.9%) and 12 women (57.1%) in the HBG, 13 men (59.1%) and 9 women (40.9%) in the PMG, and 13 men (59.1%) and 9 women (40.9%) in the CG. The mean age was 66.43 years for the HBG, 66.32 years for the PMG, and 61.73 years for the CG. The dialysis duration was 76.33 months for the HBG, 74.41 months for the PMG, and 68.82 months for the CG. Initial blood pressure measurements were 138.67/66.29 mmHg for the HBG, 141.36/69.18 mmHg for the PMG, and 143.32/69.77 mmHg for the CG (Table 1). A homogeneity test on the participants' general characteristics revealed no significant differences in sex, age, marital status, education level, religion, occupation, dialysis duration, or blood pressure.

### 2. Effects of Healing Beats application on anxiety, stress, and well-being

The homogeneity test of dependent variables revealed significant differences in stress index scores, but no significant differences in state anxiety, pulse rates, subjective stress, psychological well-being, or subjective happiness among the three groups (Table 2).

The effects of Healing Beats and preferred music listening on anxiety were analyzed, and the results are as follows. After the intervention, the anxiety scores were 39.19 ± 10.90 in the HBG, 40.59 ± 11.62 in the PMG, and 38.73 ± 9.82 in the CG, with no significant differences observed among the groups (F = 0.18, *p* = .838) (Table 2).

To assess the effects of Healing Beats and preferred music listening on pulse rate, measurements were taken nine times: before dialysis (T_0_), 1 hour after music listening (T_1h_), and at the end of dialysis (T_4h_). On the first day (D_1_), the pulse rate before dialysis (T_0_) was 68.81 ± 8.51 bpm in the HBG, 69.64 ± 12.86 bpm in PMG, and 73.14 ± 12.04 bpm in the CG (F = 0.89, *p* = .415). After 1 hour of music listening (T_1h_), the pulse rates were 66.95 ± 9.45 bpm, 69.36 ± 13.88 bpm, and 74.41 ± 9.93 bpm, respectively (F = 2.46, *p* = .094). At the end of dialysis (T_4h_), the pulse rates were 69.24 ± 9.62 bpm, 71.41 ± 14.22 bpm, and 76.27 ± 8.45 bpm, respectively (F = 2.29, *p* = .110). On the third day (D_3_), the pulse rate before dialysis (T_0_) was 69.86 ± 9.79 bpm in the HBG, 71.05 ± 10.12 bpm in the PMG, and 72.91 ± 11.51 bpm in the CG (F = 0.46, *p* = .632).

**Figure 3.**
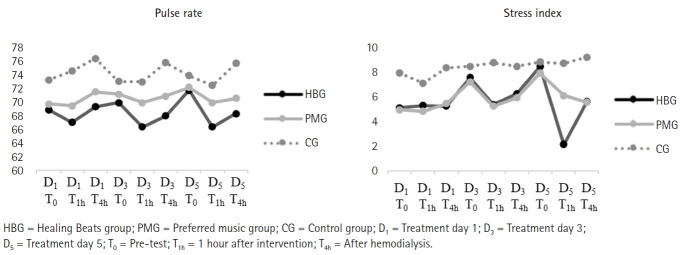
Comparison of the effects of Healing Beats on pulse rate, stress index across three groups.

After 1 hour (T_1h_), the pulse rates were 66.29 ± 7.80 bpm, 69.82 ± 11.07 bpm, and 72.86 ± 8.65 bpm, respectively (F = 2.69, *p* = .076). At the end of dialysis (T_4h_), the pulse rates were 67.90 ± 8.65 bpm, 70.77 ± 12.23 bpm, and 75.64 ± 8.10 bpm, respectively (F = 3.40, *p* = .040). On the fifth day (D_5_), the pulse rate before dialysis (T_0_) was 71.62 ± 9.91 bpm in the HBG, 72.05 ± 11.29 bpm in the PMG, and 73.73 ± 11.98 bpm in the CG (F = 0.22, *p* = .804). After 1 hour (T_1h_), the pulse rates were 66.33 ± 7.72 bpm, 69.82 ± 11.98 bpm, and 72.32 ± 10.15 bpm, respectively (F = 1.89, *p* = .160). At the end of dialysis (T_4h_), the pulse rates were 68.24 ± 9.08 bpm, 70.45 ± 11.81 bpm, and 75.55 ± 9.25 bpm, respectively (F = 2.95, *p* = .060). Repeated measures ANOVA revealed that sphericity was not satisfied (w = 0.06, *p* < .001); therefore, Wilks' lambda from multivariate testing was used to confirm the effects. The results indicated a significant difference over time (F = 4.89, *p* < .001), but no significant differences among groups (F = 2.13, *p* = .128), and no significant interaction effects between group and time (F = 0.63, *p* = .858) (Table 2, Figure 3).

The effects of Healing Beats and preferred music listening on the stress index were analyzed by measuring values 9 times: before dialysis (T_0_), 1 hour after music listening (T_1h_), and at the end of dialysis (T_4h_). Pre-intervention stress index measurements on the first day of the experiment (D_1_ T_0_), which showed significant differences during homogeneity testing, were treated as covariates. On the first day (D_1_), the stress index before dialysis (T_0_) was 5.05 ± 3.92 in the HBG, 4.91 ± 3.48 in the PMG, and 7.86 ± 3.55 in the CG (F = 4.56, *p* = .014). After 1 hour (T_1h_), the stress indices were 5.24 ± 3.52, 4.77 ± 3.93, and 7.05 ± 4.02, respectively (F = 0.45, *p* = .637). At the end of dialysis (T_4h_), the stress indices were 5.19 ± 3.67, 5.41 ± 3.95, and 8.27 ± 2.95, respectively (F = 1.44, *p* = .245). On the third day (D_3_), the stress index before dialysis (T_0_) was 7.48±2.98 in the HBG, 7.14 ± 3.08 in the PMG, and 8.41 ± 3.11 in the CG (F = 1.02, *p* = .366). After 1 hour (T_1h_), the stress indices were 5.33 ± 3.14, 5.18 ± 4.05, and 8.68 ± 2.63, respectively (F = 7.72, *p* = .001). At the end of dialysis (T_4h_), the stress indices were 6.19 ± 3.68, 5.86 ± 3.58, and 8.41 ± 3.00, respectively (F = 3.57, *p* = .034). On the fifth day (D_5_), the stress index before dialysis (T_0_) was 8.38 ± 1.94 in the HBG, 7.86 ± 2.82 in the PMG, and 8.73 ± 2.21 in the CG (F = 0.75, *p* = .477). After 1 hour (T_1h_), the stress indices were 2.10 ± 1.37, 6.05 ± 3.11, and 8.64 ± 2.30, respectively (F = 40.97, *p* < .001). At the end of dialysis (T_4h_), the stress indices were 5.57 ± 3.59, 5.50 ± 3.75, and 9.14 ± 2.17, respectively (F = 8.99, *p* < .001).

During the homogeneity test, the stress index before intervention (D_1_ T_0_), which showed significant differences among groups, was treated as a covariate. The repeated measures ANCOVA revealed that the assumption of sphericity was not satisfied (w = 0.43, *p* = .005); thus, Wilks’ lambda from multivariate testing was used to confirm the effects. Analysis of the stress index, measured 9 times, showed significant differences between groups (F = 4.36, *p* = .017). There were significant differences over time (F = 4.45, *p* = .001) and significant interaction effects between groups and time (F = 4.60, *p* < .001) (Table 2, Figure 3). Additionally, in the linear mixed effects model analysis, there was no interaction effect between groups and days (F = 2.27, *p* = .061), but the interaction effects between groups, days, and time periods were found to be significant (F = 4.58, *p* < .001). On the third day of the experiment (D_3_), the stress index in the CG remained stable, from 8.41 before dialysis (T_0_) to 8.68 1 hour after dialysis began (T_1h_). In contrast, the HBG showed a decrease from 7.48 before dialysis (T_0_) to 5.33 1 hour after music listening (T_1h_), and the PMG showed a reduction from 7.14 (T_0_) to 5.18 (T_1h_) (F = 7.72, *p* = .001). On the fifth day (D_5_), the stress index in the CG remained stable, from 8.73 before dialysis (T_0_) to 8.64 1 hour after dialysis began (T_1h_) and 9.14 4 hours after dialysis began (T_4h_). However, the HBG showed a significant reduction, from 8.38 before dialysis (T_0_) to 2.10 1 hour after music listening (T_1h_) and 5.57 4 hours after dialysis began (T_4h_). Similarly, the PMG showed a decrease, from 7.86 before dialysis (T_0_) to 6.05 (T_1h_) and 5.50 (T_4h_). These findings confirmed the significant interaction effect between groups and time (F = 4.60, *p* < .001). On the fifth day (D_5_), 1 hour after music listening (T_1h_), the stress index was highest in the CG (8.64), followed by the PMG (6.05), and lowest in the HBG (2.10), with the differences being statistically significant (F = 40.97, *p* < .001)

The effects of Healing Beats and preferred music listening on subjective stress were assessed as follows. At the end of the experiment (D_5_ T_4h_), the subjective stress scores were 4.05 ± 2.20 in the HBG, 3.95 ± 2.30 in the PMG, and 4.50 ± 2.77 in the CG, showing no significant differences among the groups (F = 0.31, *p* = .732).

The effects of Healing Beats and preferred music listening on psychological well-being were assessed as follows. At the end of the intervention (D_5_ T_4h_), the psychological well-being scores were 60.19 ± 10.00 in the HBG, 56.32 ± 7.17 in the PMG, and 58.14 ± 7.26 in the CG, with no significant differences observed (F = 1.19, *p* = .310).

For subjective well-being, At the end of the intervention (D_5_ T_4h_), the subjective well-being scores were 37.10 ± 8.89 in the HBG, 33.86 ± 9.68 in the PMG, and 37.77 ± 8.74 in the CG, with no significant differences observed (F = 1.16, *p* = .323).

For the SHS, At the end of the intervention (D_5_ T_4h_), the SHS scores were 16.48 ± 3.41 in the HBG, 15.73 ± 3.68 in the PMG, and 17.82 ± 5.18 in the CG, with no significant differences observed (F = 1.42, *p* =.251) (Table 2).

## DISCUSSION

After five intervention sessions, the results showed no significant differences in state anxiety or pulse rate among the three groups. These findings contrast with the positive effects reported in prior studies involving music interventions for hemodialysis patients [13,40] or surgical patients undergoing intraoperative music interventions [11,41]. However, they align with studies that found no significant effects of music interventions on hemodialysis patients [42]. Previous research demonstrated variability in the frequency and duration of music interventions. In this study, a total of five sessions of Healing Beats or preferred music were conducted, but no significant effects on anxiety reduction were observed. In a meta-analysis of the anxiety effect of music intervention in dialysis patients [43], six out of seven studies were conducted for more than three weeks, and this study had a total of five intervention sessions, and it is thought that there would have been a limit to improving anxiety due to a shorter intervention period than previous studies. Considering that hemodialysis patients generally experience heightened anxiety [44], stress [45], and decreased life motivation [5], the current intervention may have been insufficient to alleviate their anxiety effectively. There was no significant difference in pulse rate, which requires thorough control when physiological changes are used as indicators [46], but in hemodialysis patients with underlying diseases, I think health status and medications may have affected them.

To evaluate the effect of Healing Beats on well-being, measurements were taken before (D_1_ T_0_) and after (D_5_ T_4h_) the intervention. No significant differences in well-being were observed among the three groups after the intervention. Prior studies have shown that although well-being can be expected to have a positive effect on music intervention, it is inconsistent in the measurement method and measured in various ways, and the effect can only be confirmed for participants with low levels of well-being [13,47]. The level of well-being of the participants in this study was moderate, making it difficult to confirm significant changes. Prior studies have suggested that a positive attitude toward dialysis treatment plays an essential role in psychological well-being and health outcomes [48] and that factors such as uncertainty about illness, duration of treatment, and social support influence well-being [49]. Well-being, as a critical component of quality of life [50], can be adversely affected by the uncertainty of an incurable disease and the need for periodic medical dependence. These challenges likely limited the ability of Healing Beats or music listening to significantly enhance well-being in hemodialysis patients. In addition, it is thought to have been influenced by various external environments such as physical, psychological, and economic factors such as the patient's kidney-related symptoms, quality of social interaction, and lifestyle burden [20].

To assess the effects of Healing Beats on stress, subjective stress was measured. While no significant differences were observed in subjective stress, significant differences in the stress index were found starting from the third intervention session (D_3_). The subjective stress levels reported by the participants across all groups were moderate, and no reduction in subjective stress was observed after five intervention sessions. However, the stress index, as measured through HRV, was notably high among the participants. Starting on the third day (D_3_) of the intervention, the stress index in the Healing Beats and PMGs was lower than in the CG. By the fifth day (D_5_), significant differences in stress index were observed among the three groups, with the HBG having the lowest levels and the CG the highest. Specifically, before the fifth intervention session (D_5_), the stress index was 8.38 in the HBG, 7.86 in the PMG, and 8.73 in the CG. One hour after listening (T_1h_), the stress index decreased to 2.10 in the HBG, 6.05 in the PMG, and 8.64 in the CG, with the HBG showing the lowest levels.

There are not many studies that have conducted music intervention for dialysis patients [12] and most of the music provided is preferred music [43]. The stress index was reduced in both the HBG and the PMG mediated in this study, but the HBG induced a stable state by stimulating the parasympathetic nerve [25] showed a greater difference in stress reduction.

Healing Beats was developed for stress reduction, and previous studies on general populations reported baseline stress index scores of 4.24, 4.50, which increased to 6.02, 5.73 after exposure to stressors [20]. The level of subjective stress complained of by the subjects in the three groups was moderate, and there was no effect of reducing subjective stress after five days of experimental treatment. However, the baseline stress index of the participants in this study ranged from 4.91 to 8.73, which is a quantified based on the balance of autonomic nervous system, significantly higher than that of the general population. This aligns with the stress index levels of patients hospitalized for chemotherapy following a colorectal cancer diagnosis, which ranged from 6.59 to 7.86 [17]. These findings suggest that the stress index among hemodialysis patients is substantially higher than in the general population and comparable to that of cancer patients. After listening to Healing Beats, the stress index in the HBG significantly decreased to 2.10, a level notably lower than that of the PMG. In addition, the preceding Healing Beats study was conducted in one session [16,24,25], but in this study, it is meaningful that the repetition effect was confirmed by proceeding with five sessions.

## CONCLUSION

Hemodialysis patients exhibit a high stress index, and while Healing Beats and preferred music listening showed limited effects on reducing anxiety or improving well-being, Healing Beats was effective in significantly reducing the stress index. However, the interventions did not yield significant positive effects on subjective stress, anxiety, or well-being. Further research is needed to explore the mechanisms and optimize the implementation of Healing Beats to achieve broader psychological benefits in this population.

## Figures and Tables

**Table 1.** Baseline Characteristics of Participants across Three Groups (N = 65)

Variables	Categories	HBG (n = 21)	PMG (n = 22)	CG (n = 22)	x² or F	*p*
		
Sex	Men	9 (42.9)	13 (59.1)	13 (59.1)	1.51	.471
	Women	12 (57.1)	9 (40.9)	9 (40.9)		
Age (yr)		66.43 ± 13.11	66.32 ± 13.71	61.73 ± 13.85	69.78	.679
	≤ 40	0 (0.0)	0 (0.0)	1 (4.5)		
	41~60	6 (28.6)	7 (31.8)	8 (36.4)	-	-
	≥ 61	15 (71.4)	15 (68.2)	13 (59.1)		
Spouse	Yes	14 (66.7)	15 (68.2)	12 (54.5)	1.05	.592
	No	7 (33.3)	7 (31.8)	10 (45.5)		
Educational level^†^	≤ Elementary school	6 (28.5)	6 (27.3)	2 (9.0)	5.40	.714
	Middle or high school	8 (38.1)	10 (45.4)	12 (54.5)		
	≥ College	7 (33.3)	6 (27.3)	8 (36.4)		
Religion	Yes	12 (57.1)	10 (45.5)	9 (40.9)	1.20	.548
	No	9 (42.9)	12 (54.5)	13 (50.1)		
Job	Yes	3 (14.3)	3 (13.6)	1 (4.5)	1.35	.510
	No	18 (85.7)	19 (86.4)	21 (95.5)		
Duration of hemodialysis (mon)		76.33 ± 45.65	74.41 ± 60.34	68.82 ± 44.49	104.48	.413
Number of dialysis sessions per week		3	3	3	-	-
Comorbidities^‡^	Hypertension	12 (57.1)	14 (63.6)	12 (54.5)	-	-
	Diabetes mellitus	11 (52.4)	14 (63.6)	20 (90.9)		
	Glomerulonephritis	4 (19.0)	3 (13.6)	1 (4.5)		
	Polycystic kidney	1 (4.8)	1 (4.5)	0 (0.0)		
	Lupus	0 (0.0)	0 (0.0)	1 (4.5)		
	Renal cell carcinoma	0 (0.0)	0 (0.0)	1 (4.5)		

Values are presented as the mean ± standard deviation or n (%). HBG = Healing Beats group; PMG = Preferred music group; CG = Control group.

† The total percentage may not be exactly 100% because of rounding adjustments;

^‡^Comorbidities were recorded as multiple responses for each variable.

**Table 2.** Effects of Healing Beats Application on Anxiety, Stress, and Well-Being in the Three Groups (N = 65)

Variables	Categories		HBG (n = 21)	PMG (n = 22)	CG (n = 22)	F	*p*	F(*p*)^†^	Sphericity Test W(*p*)^‡^	F(*p*)^§^
		
STAI	D_1_ T_0_		44.10 ± 11.84	41.82 ±8.85	40.91 ±11.08	0.51	.604			
	D_5 _T_4h_		39.19 ± 10.90	40.59 ± 11.62	38.73 ±9.82	0.18	.838			
	Difference (D_5_ T_4h_-D_1_ T_0_)		−4.90 ± 10.90	−1.22 ± 7.94	−2.18 ± 10.54	1.03	.364			
Pulse rate	D_1_	T_0_	68.81 ± 8.51	69.64 ± 12.86	73.14 ± 12.04	0.89	.415	Time 4.89 (< .001)	0.06 (< .001)	
		T_1h_	66.95 ± 9.45	69.36 ± 13.88	74.41 ± 9.93	2.46	.094			
		T_4h_	69.24 ± 9.62	71.41 ± 14.22	76.27 ± 8.45	2.29	.110			
	D_3_	T_0_	69.86 ± 9.79	71.05 ± 10.12	72.91 ± 11.51	0.46	.632	Group 2.13 (.128)		
		T_1h_	66.29 ± 7.80	69.82 ± 11.07	72.86 ± 8.65	2.69	.076			
		T_4h_	67.90 ± 8.65	70.77 ± 12.23	75.64 ± 8.10	3.40	.040			
	D_5_	T_0_	71.62 ± 9.91	72.05 ± 11.29	73.73 ± 11.98	0.22	.804	Group* time 0.63 (.858)		
		T_1h_	66.33 ± 7.72	69.82 ± 11.98	72.32 ± 10.15	1.89	.160			
		T_4h_	68.24 ± 9.08	70.45 ± 11.81	75.55 ± 9.25	2.95	.060			
Stress index	D_1_	T_0_	5.05 ± 3.92	4.91 ± 3.48	7.86 ± 3.55	4.56	.014	Time 4.48 (.001)	0.43 (.005)	Group *day 2.27 (.061)
		T_1h_	5.24 ± 3.52	4.77 ± 3.93	7.05 ± 4.02	0.45	.637			
		T_4h_	5.19 ± 3.67	5.41 ± 3.95	8.27 ± 2.95	1.44	.245			
	D_3_	T_0_	7.48 ± 2.98	7.14 ± 3.08	8.41 ± 3.11	1.02	.366	Group 4.36 (.017)		Group* time 5.11 (.001)
		T_1h_	5.33 ± 3.14	5.18 ± 4.05	8.68 ± 2.63	7.72	.001			
		T_4h_	6.19 ± 3.68	5.86 ± 3.58	8.41 ± 3.00	3.57	.034			
	D_5_	T_0_	8.38 ± 1.94	7.86 ± 2.82	8.73 ± 2.21	0.75	.477	Group* time 4.60 (< .001)		Group *day *time 4.58 (< .001)
		T_1h_	2.10 ± 1.37	6.05 ± 3.11	8.64 ± 2.30	40.97	< .001			
		T_4h_	5.57 ± 3.59	5.50 ± 3.75	9.14 ± 2.17	8.99	< .001			
Subjective stress (NRS)	D_1_ T_0_		4.71 ± 2.97	3.95 ± 1.33	3.73 ± 1.72	1.28	.284			
	D_5 _T_4h_		4.05 ± 2.20	3.95 ± 2.30	4.50 ± 2.77	0.31	.732			
	Difference (D_5 _T_4h_-D_1_ T_0_)		−0.67 ± 2.29	−0.00 ± 2.49	−0.77 ± 2.43	1.93	.154			
Psychological well-being	D_1_ T_0_		60.05 ± 7.00	56.91 ± 8.70	59.45 ± 8.79	0.89	.415			
	D_5 _T_4h_		60.19 ± 10.00	56.32 ± 7.17	58.14 ± 7.26	1.19	.310			
	Difference (D_5 _T_4h_-D_1_ T_0_)		0.14 ± 7.49	−0.59 ± 4.70	−1.32 ± 5.88	0.31	.736			
Subjective well-being	D_1_ T_0_		34.24 ± 9.29	33.45 ± 9.31	35.23 ± 10.95	0.18	.838			
	D_5 _T_4h_		37.10 ± 8.89	33.86 ± 9.68	37.77 ± 8.74	1.16	.323			
	Difference (D_5 _T_4h_-D_1_ T_0_)		2.86 ± 7.24	0.41 ± 8.12	2.55 ± 8.44	0.61	.547			
Subjective happiness scale	D_1_ T_0_		16.24 ± 5.43	15.68 ± 4.03	18.32 ± 4.00	2.07	.135			
	D_5 _T_4h_		16.48 ± 3.41	15.73 ± 3.68	17.82 ± 5.18	1.42	.251			
	Difference (D_5 _T_4h_-D_1_ T_0_)		0.24 ± 3.78	0.05 ± 5.26	−0.50 ± 4.32	0.16	.855			

Values are presented as the mean ± standard deviation. HBG = Healing Beats group; PMG = Preferred music group; CG = Control group; STAI = State-trait anxiety inventory; NRS = Numeric rating scale; D_1_ = Treatment day 1; D_3_ = Treatment day 3; D_5_ = Treatment day 5; T_0_ = Pre test; T_1 h_ = 1 hour after intervention; T_4 h_ = After hemodialysis.

† Repeated measures ANCOVA;

‡ Sphericity Test by Mauchly W;

§ Linear mixed-effects model.
